# Strategies for Optimizing the Yield and Function of Recombinant Collagen in Different Expression Systems: A Review

**DOI:** 10.3390/ijms27062563

**Published:** 2026-03-11

**Authors:** Menglei Cheng, Lisheng Jiang, Zejia Zhang, Liuzhu Ji, Zhiqiang Xiong, Guangqiang Wang, Lianzhong Ai, Xinxin Liu

**Affiliations:** 1 Shanghai Engineering Research Center of Food Microbiology, School of Health Science and Engineering, University of Shanghai for Science and Technology, Shanghai 200093, China; 233442666@st.usst.edu.cn (M.C.);; 2 Department of Food Science & Technology, School of Agriculture and Biology, Shanghai Jiao Tong University, 800 Dongchuan Road, Shanghai 200240, China

**Keywords:** collagen, recombinant collagen, *Pichia pastoris*, *E. coli*, metabolic engineering, fermentation

## Abstract

Collagen is the most abundant structural and functional protein in humans and other vertebrates. It possesses remarkable biological functions and is widely used in food, cosmetics, and healthcare. Currently, mainstream animal-derived collagen materials carry risks such as viral transmission and allergic reactions. However, recombinant collagen, heterologously expressed using genetic recombination technology combined with high-density fermentation processes, offers greater biocompatibility, low immunogenicity, and consistent quality, offering promising development prospects. However, current research on recombinant collagen still faces challenges such as low yield and poor functionality. This article briefly describes the structure, types, and functions of collagen, discusses the advantages and limitations of different recombinant collagen expression systems, and highlights the strategies for improving the yield and optimizing the function of recombinant collagen, ranging from gene editing to fermentation optimization. In highlighting practical approaches to achieving high yield, we present a series of case examples to illustrate the successful application of these principles. This review aims to help researchers, engineers, and industry practitioners better understand research trends in the expression and production of recombinant collagen, and to promote its further development and commercialization across diverse application areas.

## 1. Introduction

**Figure 1:**
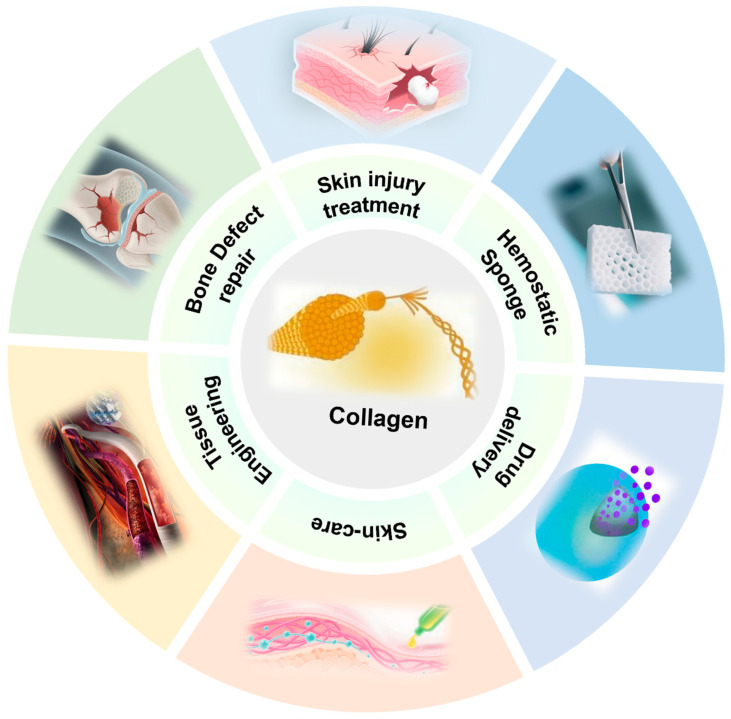
Application areas of collagen materials.

Collagen is the most abundant structural and functional protein in the human body and other animals. It is widely distributed in tissues and organs throughout the body, including skin, bones, tendons, ligaments, and cartilage. It accounts for about one-third of the total protein in the body and is equivalent to about 6% of body weight [1]. It also provides strength, durability, and flexibility to tissues [2]. At the same time, collagen, as the main component of the extracellular matrix (ECM), regulates cell behaviors such as cell proliferation, migration and differentiation by interacting with various receptors on the cell surface, thereby promoting wound healing and tissue regeneration [3]. Owing to its outstanding biological functions, biocompatibility and biodegradability, collagen has emerged as one of the most widely applied protein materials in the fields of biomaterials and regenerative medicine. Specifically, its applications cover cosmetic fillers, drug delivery systems, surgical sutures, and tissue engineering scaffolds, and other fields (Figure 1).

Currently, the collagen used in biomedicine is mainly animal-derived collagen, of which bovine collagen dominates in terms of both quality and quantity. However, the use of animal-derived collagen carries the risk of transmitting viruses, causing diseases, and allergic reactions, and it is difficult to ensure the consistency of product quality across batches. Extracting collagen from marine animal tissue can effectively avoid the pathogen contamination and allergic risks of terrestrial animal collagen, but it has disadvantages such as difficulty in extraction and high purification costs [4]. Recombinant collagen is a protein that retains key characteristics and functions of natural collagen, produced by introducing native or optimized collagen gene sequences into selected host cells, followed by culture, fermentation, separation and purification. Compared with animal-derived collagen, it boasts superior biocompatibility and low immunogenicity. Produced via microbial fermentation, it eliminates viral risks and ensures consistent batch quality under stable strains and fermentation conditions, thus holding broad application prospects in biomedicine and tissue engineering [5]. More importantly, the core advantage of recombinant technology lies in its ability to precisely produce high-purity collagen of a single subtype [6]—by targeted cloning of specific collagen genes (e.g., type I and type II), it can circumvent the problem of subtype heterogeneity inherent in naturally extracted collagen, thus meeting the specific application requirements of different scenarios.

As early as 1997, the National Dental Research Center in Maryland successfully expressed the α1 chain of mouse type IV collagen using a CHO cell line as a vector, obtaining a single-chain recombinant collagen protein [7]. Since then, scientists have used prokaryotes, yeast, plants, baculovirus, mammalian cells, etc., as expression platforms to obtain different types of recombinant collagen proteins [8]. However, the collagen proteins obtained from different expression systems have obvious differences in structure, function, and yield.

## 2. Structure and Biosynthesis of Collagen

### 2.1. Structure and Classification of Collagen

Collagen is a fibrous protein that constitutes the main structural component of the extracellular matrix, assembled from multiple tropocollagen molecules. As the fundamental structural unit of collagen, tropocollagen is formed by three left-handed helical α-polypeptide chains, which are intertwined via interchain hydrogen bonds to form a stable right-handed triple helix structure [9]. A defining feature of the tropocollagen triple helix is that each α-polypeptide chain consists of a repeating (Gly-X-Y)_n_ peptide motif. Glycine (Gly), the smallest amino acid, is the most abundant residue in this sequence, accounting for approximately 33% of the total amino acid content—this structural characteristic is essential for maintaining the compactness and stability of the triple helix. The X and Y positions can be occupied by any amino acid, but they are predominantly proline and hydroxyproline; in some cases, lysine and hydroxylysine are also present [10]. 

To date, 28 distinct collagen subtypes have been identified in animals, which are numbered with Roman numerals (I–XXVIII). These subtypes are distributed in different tissue structures and perform a variety of functions (Table 1).

Based on comprehensive criteria, including primary structure, triple helical domain length, molecular weight, triple helix interruption pattern, size and conformation of terminal domains, as well as functional specificity, these subtypes are classified into fibrillar collagens and six categories of non-fibrillar collagens [11]. Fibrillar collagens are characterized by continuous, unbroken (Gly-X-Y) repeating segments, which underpin their stable and compact triple helix conformation and enable fibril formation. In contrast, non-fibrillar collagens contain one or more interruptions in their (Gly-X-Y) sequences, leading to discontinuities in their triple helix structure [12]. These interrupted regions give rise to flexible peptide chains at the break sites, endowing non-fibrillar collagens with unique structural plasticity and specialized biological functions that differ from fibrillar collagens.

### 2.2. Biosynthetic Mechanism of Collagen

Collagen biogenesis initiates with the transcription and translation of collagen-encoding genes, which generates nascent collagen α-chains (referred to as pro-α-chains). In the endoplasmic reticulum (ER), these pro-α-chains are sequentially modified by prolyl 4-hydroxylase (P4H) and lysyl hydroxylase (LH). Specifically, these enzymes catalyze the hydroxylation of proline and lysine residues at the Yaa position within the Gly-Xaa-Yaa repeat motif, while a subset of the hydroxylated lysine residues undergoes further glycosylation [13]. Proline hydroxylation and lysine hydroxylation are essential steps for ensuring the stability of the triple helix structure and mediating the cross-linking of mature collagen in the extracellular space, respectively. Aberrant hydroxylation of nascent collagen α-chains leads to the intracellular accumulation of misfolded collagen triple helices, which in turn triggers protein-folding defects and ER stress responses [14]. Beyond the modifications by P4H and LH, pro-α-chains also require the catalytic action of prolyl 3-hydroxylase (P3H). Subsequently, the C-terminal propeptides of three pro-α-chains form disulfide bonds through the coordinated action of ER membrane lectin-like chaperones, calnexin, and the ER oxidoreductase protein disulfide isomerase (PDI) [15]. These disulfide bonds drive the trimerization of individual pro-α-chains into a triple helical conformation that extends from the C-terminus to the N-terminus [16]. Meanwhile, the collagen-specific molecular chaperone HSP47 interacts with the triple helical procollagen to prevent its local unfolding and aggregation [17]. Following HSP47-mediated stabilization, procollagen is recognized by the ER export receptor TANGO1 and transported to the Golgi apparatus. In the Golgi, HSP47 dissociates from procollagen and is recycled back to the ER via the KDEL (REDL) receptor. Concurrently, the Golgi directs the secretion of procollagen into the extracellular matrix. Finally, the N-terminal and C-terminal propeptides of procollagen are cleaved by N-proteinase and C-proteinase, respectively, yielding the mature and intact collagen molecule [18].

### 2.3. Traditional Collagen Production

Extraction from animal tissues such as porcine, bovine and fish tissues constitutes the primary source of natural collagen. The main methods for collagen extraction include acid extraction, alkaline extraction and enzymatic extraction, among others. Specifically, alkaline and acid extraction involve dissolving collagen fibers in raw materials into acidic or alkaline solutions and isolating collagen by breaking the intermolecular covalent bonds [19]. However, these methods yield a low extraction rate for tissues with highly cross-linked collagen; additionally, acidic and alkaline solutions can cause the degradation of partial collagen, which impairs the structure and biological function of the final product. Enzymatic extraction is a commonly used alternative approach: it utilizes proteases (e.g., pepsin and trypsin) to hydrolyze non-collagenous proteins, followed by separation and purification via centrifugation, filtration, salting-out and chromatography, to obtain the target collagen [20]. Nevertheless, collagen obtained by acid, alkaline or enzymatic extraction all has inherent limitations. First, the residual non-collagenous components in animal tissues (e.g., antigens, lipids and other heterogeneous proteins) may trigger immune responses in the human body, and collagen from animal tissue sources also carries the risk of disease transmission. More importantly, collagen in animal tissues does not exist as a single subtype but as a mixture of multiple subtypes (e.g., type I and type II) [21], making it difficult to obtain a single purified collagen subtype via direct extraction from animal tissues.

Additionally, traditional animal tissue collagen extraction relies heavily on animal husbandry, consuming large amounts of resources (land, feed, water, etc.), with high treatment costs for wastewater and animal by-products from breeding and slaughter. Extensive use of acids, alkalis and other chemical reagents in extraction further increases costs and causes severe environmental pollution. In contrast, industrial wastewater and solid waste from large-scale recombinant collagen fermentation are more centrally and efficiently treated, and precision fermentation is expected to enable more sustainable and cost-effective production [22].

### 2.4. Regulations and Standards for Recombinant Collagen

Recombinant collagen is synthesized by using genetic recombination technology to introduce the target gene into the host cell at the molecular level and using the host cell’s transcription and translation system. Recombinant collagen is prepared from human-derived gene fragments via heterologous expression technology, enabling the acquisition of a single purified collagen subtype. It has the advantages of high biocompatibility and low immunogenicity. With the rapid development of molecular biotechnology, protein expression technology has become increasingly mature and standardized, prompting scientific researchers to gradually shift their research focus from animal-derived collagen to recombinant collagen expression [23]. 

At present, recombinant collagen is regulated as a biomaterial, medical device, or biological product in major countries and regions around the world. In China, in accordance with the *Guideline on Evaluation of Recombinant Humanized Collagen Raw Materials* [24] and the industrial standard *Recombinant Collagen* (YY/T 1849-2022) [25] issued by the National Medical Products Administration (NMPA), the core regulatory requirements for recombinant collagen can be summarized as follows: the functional completeness of recombinant collagen is comprehensively evaluated from the perspectives of sequence origin, structural characteristics (including primary and secondary structures, as well as the presence or absence of a triple-helical structure), degree of post-translational modifications, and biological activity. Specifically, standardized methods such as circular dichroism (CD), differential scanning calorimetry (DSC), sodium dodecyl sulfate-polyacrylamide gel electrophoresis (SDS-PAGE), and size-exclusion chromatography coupled with multi-angle light scattering (SEC-MALS) are adopted to determine its specific structural parameters, including triple-helical properties (thermal stability Tm, helix content, molecular uniformity, etc.). The hydroxyproline content is measured via acid hydrolysis combined with high-performance liquid chromatography (HPLC) or amino acid analysis, so as to determine the levels of hydroxylation and glycosylation, as well as the presence of abnormal modifications such as deamidation and truncation. Biological activity is mainly evaluated through standardized assays of cell adhesion, proliferation, cytotoxicity, and specific animal experiments.

## 3. Recombinant Collagen Expression System

**Figure 2:**
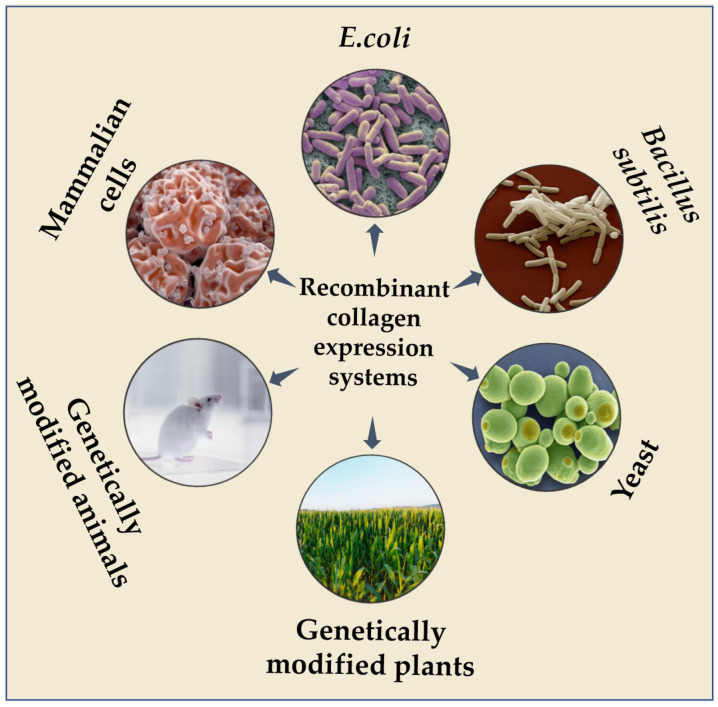
Recombinant collagen expression systems.

According to the type of host cell, recombinant collagen expression systems can be divided into two categories: eukaryotic and prokaryotic. Eukaryotic expression systems mainly include yeast, genetically modified plants, genetically modified animals and mammalian cells; prokaryotic expression systems mainly include *E. coli* and *Bacillus subtilis* (Figure 2).

Different host expression systems have different characteristics. Among them, the collagen expressed by mammalian cells has a higher similarity to natural human collagen and a higher yield. However, the construction of the mammalian cell expression system is relatively complex, the culture cost is high, and there is a risk of viral infection during culture. The recombinant proteins produced via plant cell expression systems are free from the risk of viral infection. However, low yield remains a key drawback of plant cell-based recombinant collagen production, making it hard to satisfy market needs [26]. The insect baculovirus expression vector system can also perform the post-translational modification and processing of recombinant collagen, but it still has the disadvantages of a relatively complex operation process, long culture cycle, and low yield, and further research and improvement are still needed [27].The fermentation expression of recombinant collagen using microorganisms such as *E. coli* or yeast has a high expression yield, low cost per unit collagen expression, simple operation, short preparation cycle, and excellent potential for large-scale industrial production. Thus, a substantial number of studies have concentrated on utilizing *E. coli* or yeast for recombinant collagen research and production.

### 3.1. Escherichia coli Expression System

The prokaryotic expression system for recombinant collagen is mainly based on the *E. coli* expression system. As one of the earliest-established and most extensively utilized recombinant protein expression systems, *E. coli* features a well-characterized genetic background, low fermentation costs, short production cycles, and high efficiency. It can quickly produce exogenous proteins on a large scale and has the potential for large-scale production of exogenous proteins [28].

Many research teams have successfully expressed various recombinant collagens using the *E. coli* expression system [29]. Chang et al. expressed recombinant type II collagen in *E. coli* via high-density fermentation, and through the optimization of culture and induction conditions, ultimately obtained a yield of up to 13.2 g/L [30]. In recent years, Ling et al. selected a specific fragment of human type III collagen, which was then tandemly repeated 18 times, to express a novel recombinant collagen variant in *E. coli*. This recombinant collagen exhibits excellent biocompatibility and cell adhesion properties [31].

However, *E. coli* does not encode proline hydroxylase, the indispensable enzyme responsible for the post-translational hydroxylation of proline residues, which results in its inability to hydroxylate the recombinantly expressed collagen. This deficiency makes it difficult for the produced collagen to effectively form a triple helix structure. To address this limitation, Shi et al. co-expressed full-length recombinant type III collagen and proline hydroxylase A085R in *E. coli*. They utilized proline hydroxylase to hydroxylate the proline residues in the intracellularly expressed collagen, ultimately obtaining recombinant collagen with a proline hydroxylation percentage of 73% [32]. The yield of hydroxylated collagen synthesized by *E. coli* is mostly low, and the *E. coli* expression system may also carry the risk of endotoxins. In addition, recombinant proteins are usually expressed intracellularly in *E. coli* and require extraction through steps such as cell disruption, separation, and purification—this is a relatively complicated process. These problems require further scientific research to solve.

### 3.2. Yeast Expression System

Yeast, as a eukaryotic organism, is capable of mediating post-translational modification and secreting recombinant proteins—key advantages for its application in protein expression. To date, researchers have developed numerous yeast species into engineered expression systems for recombinant protein production, including *Pichia pastoris*, *Hansenula* spp., and *Saccharomyces cerevisiae*, and have achieved successful expression of a broad range of target proteins [33]. Among these yeast expression systems, *Pichia pastoris* has emerged as a commonly used host strain for recombinant collagen expression. As a successful protein expression system, *Pichia pastoris* is also a “generally recognized as safe” (GRAS) microorganism and is widely used in the fields of industrial enzymes and biopharmaceuticals. In addition to recombinant collagen, it has also been used to produce more than 500 pharmaceutical proteins and more than 1000 recombinant proteins [34]. The *Pichia pastoris* expression system has the characteristics of being easy to culture as a unicellular microorganism and being able to modify recombinant proteins as a eukaryotic organism. *Pichia pastoris* has a strong preference for aerobic growth, which can achieve high-density cell culture and is conducive to large-scale industrial production. *Pichia pastoris* can secrete and express exogenous proteins at high levels, and the accumulation of fermentation products will not cause toxic side effects and thus affect the yield. In addition, *Pichia pastoris* secretes very little endogen protein into the culture medium, which is convenient for purification [35]. 

The core mechanism of recombinant collagen expression in the *Pichia pastoris* system starts with the integration of the collagen expression cassette into the *Pichia pastoris* genome, usually at the AOX1 locus. This allows the target gene to be regulated by the strong AOX1 promoter, enabling highly efficient transcription under methanol induction. To overcome translational bottlenecks, the collagen gene is generally codon-optimized to match the codon usage frequency of *Pichia pastoris*. In addition, recombinant collagen is usually expressed in a secreted form using signal peptides such as the α-mating factor [36]. The signal peptide directs the nascent polypeptide into the ER and is cleaved by the Kex2 protease during transport through the Golgi apparatus, ultimately releasing the correctly processed mature collagen into the fermentation supernatant.

In 2001, Werten et al. selected a collagen fragment, repeated it four times, and expressed it in *Pichia pastoris*, ultimately obtaining a recombinant collagen protein with a yield of 6 g/L [37]. In 2006, Kawaguchi et al. showed that the yield of recombinant collagen expressed in *Pichia pastoris* is related to the molecular weight and the length of the peptide chain [38]. In 2014, Li et al. used *Pichia pastoris* GS115 to express the human type III collagen α1 chain without the N-terminal and C-terminal domains and obtained a recombinant collagen protein with a yield of approximately 4.68 g/L after high-density fermentation [39]. 

Prolyl-4-hydroxylase (P4H) is an ER enzyme. In vertebrates, this enzyme exists as an α_2_β_2_ tetramer, which hydroxylates proline residues on procollagen chains to form hydroxyproline. The formation of the collagen triple helix is critically dependent on the hydroxylation of proline. The core strategy for overcoming the challenge of prolyl hydroxylation in collagen expression using the *Pichia pastoris* system is to co-express a heterologous P4H and optimize the activity of this enzyme. In 2015, He et al. used the human-derived P4H gene to perform hydroxylation modification on recombinant type III collagen. After fermentation, the same methods of amino acid composition analysis and liquid chromatography-tandem mass spectrometry (LC-MS/MS) were employed. The results showed that co-expressing human P4H significantly increased the hydroxylation rate of recombinant collagen, with the highest rate reaching 65.52% [40]. Considering that the use of identical promoters to drive the expression of two genes may affect the expression level of each individual gene, Shi employed the *Pichia pastoris* expression system and constructed a co-expression system using heterologous promoters. Specifically, the recombinant human collagen gene and the P4H gene were controlled by the AOX1 and FLD1 promoters, respectively. As a strong inducible promoter, FLD1 is also induced by methanol. The results showed that the hydroxylation level of proline was further improved in the system regulated by heterologous promoters, with a hydroxylation ratio of 71.16% [41].

## 4. High-Yield Strategies for Recombinant Collagen

Recombinant collagen holds enormous application potential in terms of biocompatibility and functionality. Due to its high sequence homology with human collagen, recombinant collagen exhibits extremely low immunogenicity [42]; moreover, its amino acid composition and triple helix structure can be precisely tailored via genetic engineering, thereby enhancing its capacity to promote cell adhesion, proliferation and matrix remodeling. In addition, recombinant collagen can circumvent the inherent risks of pathogen contamination and batch-to-batch variability associated with animal-derived collagen, a feature that is crucial for its clinical translation and industrial application [43]. To achieve higher yields of recombinant collagen, researchers have primarily opted for microbial fermentation processes for large-scale industrial production. To further increase recombinant protein yields, researchers have utilized genetic engineering and fermentation engineering techniques, optimizing expression elements and regulating metabolic networks through genetic modification of engineered bacteria, and achieving significant increases in target protein production through high-density fermentation (Table 2).

### 4.1. Promoter Selection and Induction Condition Optimization

Promoters are key elements of gene expression and play an important role in initiating gene transcription. Highly active promoters initiate gene expression more frequently and produce more mRNA, and the level of mRNA expression is generally positively correlated with protein expression. Selecting an appropriate promoter is key to efficiently expressing recombinant proteins. Promoters can be divided into inducible promoters that require inducers and constitutive promoters that do not.

The AOX1 promoter, derived from the *Pichia pastoris* alcohol oxidase gene, is a widely used inducible promoter that has been successfully commercialized. As one of the strongest promoters in the *Pichia pastoris* expression system, it enables rapid transcription of downstream genes under sufficient methanol induction [44]. This promoter is strictly induced by methanol and repressed by glucose and glycerol. In practical applications, glycerol or glucose is usually used for cell biomass accumulation in the early stage of fermentation, followed by switching to methanol as both the carbon source and inducer to trigger protein expression. In addition to the AOX1 promoter, other common methanol-inducible promoters in *Pichia pastoris* include DAS1, DAS2, FLD1, and FDH1. The GAP promoter (P_GAP_) from the glyceraldehyde-3-phosphate dehydrogenase gene is the most frequently used constitutive promoter, exhibiting high transcription strength in both glucose and methanol media.

Industrial fermentation of recombinant *E. coli* mainly adopts nutrient source induction, temperature induction, Isopropyl β-D-1-Thiogalactopyranoside (IPTG) or lactose induction, among which the majority of *E. coli* expression system strains used on an industrial scale are IPTG-inducible, and commonly used promoters include T7 promoter, tac promoter and lac promoter. When using IPTG to induce recombinant *E. coli* to express recombinant proteins, attention should be paid to the concentration of IPTG used. If the concentration is too low, the promoter activation will be weakened, resulting in a decrease in the production level of the expressed recombinant protein; while adding too high a concentration of IPTG will have a toxic effect on the cells, resulting in a decrease in cell growth rate and even accelerated cell death, which will also lead to a decrease in the production of recombinant protein [45]. However, due to the toxicity of IPTG and the high price of IPTG itself, lactose is often used as an inducer instead of IPTG in large-scale fermentation. The ratio of lactose to other carbon sources is also a key factor affecting the yield of recombinant protein. If the lactose concentration is too low, carbon sources such as glycerol will repress or inhibit the promoter, making it impossible for the promoter to start or the starting strength to be insufficient, which can easily lead to no expression or low-level expression; and if the lactose concentration is too high, the bacteria will only use lactose, resulting in a waste of carbon sources [46]. 

Ma et al. used *Pichia pastoris* to express recombinant collagen protein ColP2. They selected four endogenous promoters (P_AOX1_, P_FLD1_, P_GCW14_ and P_TEF1_) for comparative expression of ColP2, a recombinant human type III collagen with a triple helix structure, aiming to improve its yield. The experimental results showed that the yield reached 0.45 g/L when P_AOX1_ was used, which was significantly higher than that of the other tested promoters. Finally, P_AOX1_ was selected for their subsequent experiments [47]. 

In summary, rational selection of promoters and optimization of induction conditions are critical for improving transcription efficiency and increasing the yield of recombinant collagen.

### 4.2. Effect of Gene Copy Number on Recombinant Collagen

Gene expression has a dosage effect. In theory, increasing the copy number of a gene will increase the expression of the gene within a certain range. To increase the yield of recombinant collagen, the copy number of the corresponding gene of recombinant collagen can be increased.

Cai et al. intercepted the 2731 to 3417 bases of the gene sequence of the α1 chain of human type III collagen as an expression fragment and constructed a recombinant collagen four-copy vector pPIC9K-COL3-4 based on this gene. They expressed it in *Pichia pastoris* and obtained a recombinant collagen yield of 1.98 g/L in a 5 L fermenter [48]. 

However, it is not the case that the higher the copy number, the higher the protein expression. Zhu et al. used *Pichia pastoris* to express porcine insulin. When the gene copy number was 6, the yield was high, but when the gene copy number was further increased, the yield decreased [49]. 

Excessive increase in the expression of exogenous recombinant protein will consume a large number of protein chaperones in the ER, increase the synthesis pressure of the ER, and ultimately interfere with the correct folding of the cell’s own proteins, causing ER stress, thus resulting in decreased expression of the target protein [50].

In conclusion, adjusting the gene copy number within an appropriate range can effectively improve the yield of recombinant collagen, while excessive increase in copy number will lead to a decrease in yield due to ER stress.

### 4.3. Co-Expression of Molecular Chaperones Alleviates ER Stress

Overexpression of foreign proteins in engineered strains imposes a metabolic burden and triggers ER stress [51]. When unfolded proteins accumulate inside the ER, the molecular chaperone KAR2 detaches from IRE1 and binds to the misfolded proteins to assist their correct folding. The liberated IRE1 then forms oligomers and undergoes autophosphorylation, which activates its endonuclease activity. This activity promotes the splicing of HAC1 mRNA, enabling the synthesis of the functional transcription factor HAC1p. HAC1p subsequently translocates into the nucleus and upregulates the transcription of key UPR-related genes such as ERO1 and PDI [52].

Wang et al. [53] constructed several *Pichia pastoris* engineering strains with different copy numbers expressing recombinant type III human-like collagen α1 (hlCOLIII). By analyzing the expression level of recombinant collagen and detecting ER stress in strains with different copy numbers, they confirmed that multi-copy strains could effectively increase the expression of hlCOLIII, but caused a certain upregulation of the UPR, indicating the occurrence of ER stress. To alleviate ER stress, the authors selected seven molecular chaperones for co-expression: LHS1, BMH2, HAC1, AFT1, KEX2, Ssa4 and Kar2. Among them, the HAC1 chaperone was derived from three sources: *P. pastoris* (PpHAC1), *S. cerevisiae* (ScHAC1), and Homo sapiens (HsHAC1). The results showed that, compared with the control strain, the engineered strains co-expressing PpHAC1 and HsHAC1 increased the yield of hlCOLIII by 15.7% and 10.8%, respectively. Among them, co-expression of PpHAC1 exhibited the best effect, with the highest hlCOLIII yield reaching 0.81 g/L. In addition, RT-PCR results showed that the transcription levels of other UPR-related genes were decreased except for the HAC1 gene, indicating that ER stress was alleviated. The results of this study demonstrated that co-expression of molecular chaperones can effectively relieve ER stress and improve the yield of recombinant collagen.

In conclusion, co-expression of appropriate molecular chaperones can effectively alleviate ER stress caused by overexpression of recombinant collagen, thereby improving protein folding efficiency and increasing the yield of recombinant collagen.

### 4.4. Modification of Key Genes to Enhance the Yield of Recombinant Collagen

Genetic engineering technology is used to analyze and regulate the metabolic network of engineered bacteria. By knocking out or overexpressing genes in certain metabolic pathways, the engineered bacteria can achieve efficient utilization of substances and energy, reduce the metabolic pressure of engineered bacteria in expressing exogenous recombinant proteins, and reduce the generation of harmful by-products that affect cell growth and recombinant protein expression, ultimately achieving high-yield expression of recombinant proteins.

During the culture of recombinant *E. coli*, glucose is the most widely used carbon source in the culture medium. It mainly enters the cell through the phosphotransferase system (PTS) and participates in metabolism. When the excess carbon source metabolic flow absorbed by the cell passes through pyruvate, if it exceeds the processing capacity of the tricarboxylic acid cycle, acetic acid will be produced. When the acetic acid concentration is too high, it will inhibit cell growth and lead to a decrease in the production of recombinant protein [54]. 

**Table 2:** Strategies for optimizing the yield of recombinant proteins.

Expression System	Protein Type	Yield Optimization Strategy	Yield	Hydroxylation Modification (Present/Absent)	Triple-Helical Structure (Present/Absent)	References
*Pichia pastoris*	Recombinant type III collagen polypeptide	Screening for the optimal promoter	0.45 g/L	present	present	[47]
*Pichia pastoris*	Recombinant type III collagen polypeptide	Improving mRNA copy numbers	1.98 g/L	insufficient	absent	[48]
*Pichia pastoris*	Type III human-like collagen	Co-expression of Molecular Chaperones Alleviates ER Stress	0.81 g/L	absent	absent	[53]
*Escherichia coli*	Human-like collagen	Knockout of *ptsG* gene	7.15 g/L	absent	absent	[55]
*Pichia pastoris*	Human COL2 fragments	Knockout of *pep4* gene	3.04 g/L	present	present	[56]
*Pichia pastoris*	Silk fibroin proteins	Overexpression of serine *SHM2*, *GRS* and tRNA^Gly^ genes	11.39 g/L 9.48 g/L	/	/	[57]
*Escherichia coli*	Human-like collagen III	Optimize fermentation process parameters	9.68 g/L	absent	absent	[58]
*Pichia pastoris*	Recombinant type III collagen polypeptide	Fermentation parameter optimization; Feeding strategy optimization	19.49 g/L	insufficient	dimeric-structured	[59]

Luo et al. constructed *Escherichia coli BL21* strains expressing Human-like collagen (HLC) and regulated its glucose absorption and metabolism capacity by knocking out the *ptsG* gene in its PTS, reducing its carbon source metabolic flow entering the glycolysis pathway and making the carbon source metabolic flow entering the tricarboxylic acid cycle roughly balanced. Ultimately, compared with the original strain, the accumulation of acetic acid was reduced by about 42%, and the production of recombinant collagen increased from 5.6 g/L to 7.15 g/L [55].

Certain proteases inherent in engineered bacteria can cause degradation of the target protein, thus affecting yield. Therefore, systematically knocking out certain key protease genes can reduce target protein degradation and thus increase yield without affecting the normal life activities of the engineered bacteria. Wang et al. used *Pichia pastoris* to express type II collagen. Previous research revealed that the Pichia protease Pep4 significantly degrades the target protein. Therefore, by knocking out the Pep4 gene in their engineered bacteria and using fed-batch fermentation, they increased the target collagen yield to 3.04 g/L [56].

By enhancing the supply and transport capacity of key substances in the engineered bacteria expressing the target protein, its yield can also be effectively increased. Tian et al. used *Pichia pastoris* to express two silk fibroin proteins, in which glycine accounted for more than 40%. Therefore, they overexpressed the serine hydroxymethyltransferase (*Shm2*), glycyl-tRNA synthetase (*Grs*), and tRNA^Gly^ genes of their engineered bacteria to enhance the synthesis and transport capacity of glycine, resulting in a significant increase in the final yield of their target protein [57]. The glycine content of recombinant collagen is similar to that of silk fibroin, also as high as 1/3. Therefore, this modification strategy has reference value for increasing the yield of recombinant collagen expressed in *Pichia pastoris*.

In conclusion, rational modification of key genes related to metabolic regulation, protease degradation and amino acid supply can effectively reduce metabolic burden and protein degradation, thereby significantly improving the yield of recombinant collagen.

### 4.5. High-Density Fermentation

High-density fermentation, also known as high-cell density fermentation, refers to a fermentation technique that, under specific culture conditions and systems, significantly increases the density of the bacterial cells compared to conventional fermentation, thereby producing more or more efficient target products. High-density fermentation has become a key fermentation process for pilot production in biotechnology. It offers stable processes, short fermentation cycles, and significantly increases both bacterial density and recombinant protein yield compared to non-high-density fermentation. It also reduces production costs while improving efficiency. Factors influencing the expression of exogenous proteins in shake flasks and fermenters using high-density fermentation primarily include culture medium composition, temperature, pH, dissolved oxygen levels, and fed-batch dosing strategies.

Zhang et al. used *E. coli* BL21 to produce recombinant type III collagen by high-density fermentation. They used Plackett–Burman and Box–Behnken design to optimize the fermentation process parameters and determined the optimal induction time of 3.2 h, seed age of 12.6 h, and pH of 6.7. They successfully increased the recombinant collagen yield from the original 5.36 g/L to 9.68 g/L [58]. 

Liu et al. used *Pichia pastoris* to produce recombinant humanized collagen. They used the induction pH, temperature, and methanol addition as design variables and the recombinant collagen yield as the response value. They optimized the fermentation conditions of *Pichia pastoris* by response surface methodology and determined the optimal values of the three factors as follows: induction pH of 4.95, temperature of 28.16 °C, and methanol addition of 2.16%/24 h. Finally, high-density fermentation was carried out in a fully automatic 12.5 L fermenter, and a recombinant collagen yield of 19.49 g/L was obtained, which is at the leading level in the industry [59].

In conclusion, high-density fermentation combined with systematic optimization of fermentation parameters is an effective strategy to significantly improve the yield of recombinant collagen.

## 5. Structure and Function Optimization of Recombinant Collagen

In recent years, although notable progress has been made in the research and preparation of recombinant collagen, it still faces tremendous challenges in terms of its biological activity and functional properties, in addition to yield. Because the structure of recombinant collagen is the material basis for its function, in order to obtain recombinant collagen with higher activity and function, precise sequence design, post-translational modification, and supramolecular assembly can be used to manipulate its mechanical properties, bioactivity, and degradation behavior to meet diverse needs such as skin tissue repair and drug delivery. The key to collagen’s physiological functions lies in its primary structure, specifically its amino acid sequence [60]. For example, human type III collagen is widely distributed in extensible connective tissues such as the skin, internal organs, and the vascular system, playing a vital role in wound healing, collagen fibrillogenesis, and normal cardiovascular development. Certain specific amino acid sequences and charged residues within the α1 chain of type III collagen are key to its unique structure and function [61]. By designing and modifying the amino acid sequence of recombinant collagen to optimize its structure, novel recombinant collagen with stronger bioactivity and functional properties can be obtained.

Chen et al. found that the Gly489-Gly510 fragment in the triple helix region of the α1 chain of type III collagen contains multiple polar charged residues with long side chains and Glu-Lys-Gly and Glu-Arg-Gly triplets, and observed a variety of inter-chain hydrogen bonds and inter-helical hydrogen bonds in its structure that help stabilize its triple helix structure, and found that the Gly489-Gly501 fragment showed a significant bend of 15.12°, indicating that it has strong flexibility. In order to explore the impact of the above special structure on the function of collagen, the authors compared the cell adhesion function of various collagens and finally determined that the recombinant type III collagen T16 (Gly483-Pro512, repeated in series 16 times) they constructed has very strong cell adhesion activity. This study shows that the cell adhesion activity of recombinant collagen is very important. It is related to its specific sequence and the special crystal structure it possesses [62].

The sequence selection, design, and repetition strategy of recombinant humanized collagen also exert a significant impact on its structure and function. Studies have found that 16 amino acids (KPGPRGGQRGPTGPRGE) in the HEPV fragment of the α1 chain of type V collagen have a negative regulatory function on pathological angiogenesis. To study the effect of different repeat numbers on collagen function, Yan et al. synthesized this collagen hexadecapeptide (1601) by chemical synthesis and used *Pichia pastoris* to recombinantly express collagens 1605 and 1610 with 5 and 10 repeats, respectively. CD analysis showed that the secondary structures of proteins 1605 and 1610 mainly exhibit β-reversed folds and random coils. These flexible and stable random coil structures are important regions for functional realization and conceptual transformation within protein molecules. Finally, the authors used molecular docking and cell migration and invasion experiments to prove that repeated recombinant collagen 1605 and 1610 had a more significant effect on inhibiting excessive proliferation of blood vessels [63].

The structure and function of collagen are closely related to its post-translational modification, especially proline hydroxylation. Human collagen undergoes two types of proline hydroxylation: one is the rare hydroxylation by prolyl 3-hydroxylase (P3Hs) to 3-hydroxyproline, and the other is the more common hydroxylation by prolyl 4-hydroxylase (P4Hs) to 4-hydroxyproline [64]. It is often difficult to achieve post-translational modification of collagen by recombinant expression using microorganisms, especially prokaryotic systems such as *E. coli*, thus affecting its biological function. To investigate the effects of proline hydroxylation on recombinant collagen, Zhu et al. co-expressed a 168-371AA fragment (named 3-3), containing multiple biological functional motifs in the triple helical region of the α1 chain of type III collagen, with three prolyl hydroxylases, BaP4H, DsP4H, and L593, using *E. coli*. Characterization of its structure by CD spectroscopy revealed that 3-3 failed to form a triple helical structure, while 3-3 (BaP4H) and 3-3 (L593) modified by proline hydroxylase both formed triple helical structures, and 3-3 (DsP4H) lacked a characteristic triple helical structure; thermal stability experiments demonstrated that hydroxylation enhanced its thermal stability to varying degrees. Cell adhesion results showed that the triple helical structure of recombinant humanized type III collagen was closely related to its adhesion function. This study confirms that proline hydroxylation modification is the basis for the formation of the triple helical structure of recombinant collagen, as well as an important characteristic distinguishing it from other collagen polypeptides; moreover, the presence or absence of the triple helical structure exerts a profound impact on its thermal stability and adhesion function [65]. 

Through optimization and screening of the target recombinant collagen sequence, precise removal of unstable amino acid residues and tandem expression of specific peptide fragments with high stability can significantly improve the stability and bioactivity of recombinant collagen in terms of molecular structure, aggregation state, and resistance to environmental stress. Based on the human type III collagen (hCOL3) sequence, Deng et al. screened and obtained a region (G1089-A1172) with high cell adhesion activity. They subsequently deleted the “GIKGHR” sequence containing cleavage sites and repeated the remaining sequence six times to obtain the Y197 variant. Further removal of the high-risk cleavage sites “RGPVGP” and the N-terminal “GPRG” finally yielded the improved recombinant collagen variant Y326. Y326 exhibits a triple-helix structure similar to native hCOL3 and shows strong resistance to trypsin digestion. The researchers also conducted a comprehensive stability evaluation of Y326, including in vitro storage stability and in vivo pharmacokinetic studies. The results confirmed its favorable storage stability under various storage conditions. Moreover, excellent bioactivity was verified via multiple cellular and animal experiments, such as promoting cell adhesion, migration, and proliferation [66].

## 6. Conclusions and Prospects

Although recombinant collagen has demonstrated tremendous potential in terms of biocompatibility and functionality, further optimizing its production process to ensure large-scale, stable, high-quality, and high-quantity production remains a key research direction and faces numerous technical challenges. While there are currently a variety of expression systems for recombinant collagen, large-scale microbial fermentation, primarily involving *E. coli* and *Pichia pastoris* expression systems, is the most promising production method, considering factors such as cost, technical difficulty, and yield. Furthermore, to further increase recombinant protein production, researchers are utilizing genetic engineering and fermentation engineering technologies to genetically modify engineered bacteria, optimize their expression elements and regulate metabolic networks, and ultimately achieve a significant increase in target protein production through high-density fermentation. These yield optimization strategies include selecting the optimal promoter (such as *Pichia pastoris* AOX1 or *E. coli* T7/Tac) and fine-tuning induction conditions (such as methanol and lactose concentrations); optimizing gene copy number; manipulating metabolic networks to modify carbon source utilization pathways (such as knocking out the *ptsG* gene) to reduce byproducts such as acetate and alleviate metabolic stress; employing a dual-promoter strategy to enhance expression; regulating related genes to enhance the supply and transport of key target substances; knocking out protease genes (such as Pep4) to reduce degradation; and co-expressing molecular chaperones to promote folding and secretion. Fermentation optimization can utilize high-density fermentation processes and integrate response surface optimization of culture medium, pH, dissolved oxygen, and feeding strategies to achieve significant yield increases.

Although recombinant collagen has achieved certain progress, it still faces challenges such as the requirements for higher functionality and greater yield. With the continued advancement of genetic engineering, synthetic biology, cell culture technology, and fermentation engineering, the integration of synthetic biology with artificial intelligence to develop intelligent dynamic control systems will further coordinate internal host modifications with external processes, ultimately achieving efficient and green production of recombinant collagen.

## Figures and Tables

**Table 1:** Types, distribution, subunit composition and functions of collagen [9].

Type	Distribution Subunit	Composition	Function
I	Skin, bones, tendons, cornea, organs, and blood vessels	α1[I]_2_α2[I]	Mutations can lead to osteoporosis, tooth deformities, bluish sclera, thinning skin, weak tendons, and hearing loss. It binds to bone morphogenetic protein-2 and transforming growth factor P, promoting cartilage development
II	Cartilage	α1[II]_3_	Binds to bone morphogenetic protein-2 and transforming factor P, which promotes the development of cartilage
III	Reticular fibers, blood vessels, skin, uterus, and intestines	α1[III]_3_	Mutations in this gene cause Ehlers–Danlos syndrome
IV	Basement membrane and capillaries	α1[IV]_2_α2[IV], α3[IV]α4[IV]α5[IV], α5[IV]_2_α6[IV]	Support structure of cells and tissues, inhibit angiogenesis and tumor growth
V	Cells, bones, skin, placenta, cornea, hair	α1[V]_3_, α1[V]_2_α2[V], α1[V]α2[V]α3[V]	Neural development and regeneration, mutations in which cause Ehlers–Danlos syndrome
VI	Skin, bones, blood vessels, cartilage, and cornea	α1[VI]α2[VI]α3[VI] α1[VI]α2[VI]α4[VI]	Support structure of cells and tissues, muscle function
VII	Mucous membranes, bladder, skin, amniotic fluid, and umbilical cord	α1[VII]_2_α2[VII]	Mutations that cause epidermolysis bullosa
VIII	Heart, skin, kidneys, brain, bones, blood vessels, and cartilage	α1[VIII]_3_, α2[VIII]_3_, α1[VIII]_2_α2[VIII]	Serve as a support structure for cells and tissues
IX	Cornea, cartilage	α1[IX]α2[IX]α3[IX]	Maintain the integrity and stability of the extracellular matrix and regulate the formation process of collagen
X	Cartilage	α1[X]_3_	Acts as a support structure for cells and tissues, promoting cartilage development
XI	Cartilage and intervertebral disc	α1[IX]α2[IX]α3[IX]	Promote cartilage development
XII	Cartilage, skin, tendons	α1[X]_3_	Maintain the integrity and stability of the extracellular matrix and tissues, and regulate the formation of collagen
XIII	Skeletal muscle, eyes, heart, endothelial cells, and skin	α1[XI]α2[XI]α3[XI]	Transmembrane collagen associated with neuromuscular junction development
XIV	Blood vessels, nerves, eyes, bones, tendons, cartilage, and skin	α1[XII]_3_	Maintain the integrity and stability of the extracellular matrix and tissues, and regulate the formation of collagen
XV	Capillaries, heart, ovaries, skin, testicles, kidneys, and placenta	α1[XIII]_3_	Inhibits angiogenesis and tumor growth
XVI	Skin, heart, smooth muscle, and kidneys	α1[XIV]_3_	Maintain the integrity and stability of the extracellular matrix and regulate the formation process of collagen
XVII	Skin	α1[XVII]_3_	Mutations in this gene cause epidermolysis bullosa, inhibit angiogenesis and tumor growth, signaling molecule receptors, and maintain kidney morphology
XVIII	Kidneys, liver, and lungs	α1[XVIII]_3_	Mutations in this gene cause epidermolysis bullosa, inhibit angiogenesis and tumor growth, signaling molecule receptors, and maintain kidney morphology
XIX	Skin, liver, kidneys, spleen, placenta, and prostate	α1[XIX]3	Regulates the collagen formation process
XX	Corneal epithelium	α1[XX]_3_	Maintain the integrity and stability of the extracellular matrix and regulate the formation process of collagen
XXI	Stomach, heart, kidneys, placenta, skeletal muscle, and blood vessels	α1[XXI]_3_	Extracellular matrix component of the blood vessel wall, secreted by smooth muscle cells
XXII	Organizational connections	α1[XXII]_3_	Structurally and functionally independent aggregates of cartilage matrix that are integrated with the extracellular matrix of cartilage fibers
XXIII	Metastatic carcinoma cells	α1[XXIII]_3_	Essential for tissue proliferation, key structure of the extracellular matrix
XXIV	Bones and cornea	α1[XXIV]_3_	Participates in the formation of bones, bone mineralization and regulation of bone homeostasis
XXV	Eyes, heart, brain, and testicles	α1[XXV]_3_	Plays a role in neuromuscular development and cancer metastasis and has been implicated in Alzheimer’s disease
XXVI	Testicles and ovaries	α1[XXVI]_3_	Associated with thyroid cancer
XXVII	Cartilage, dermis, cornea, retina, and heart arteries	α1[XXVII]_3_	Involved in notochord morphogenesis, vertebral mineralization, and post-embryonic axial growth
XXVIII	Renal tubular epithelial cells	α1[XXVIII]_3_	Associated with kidney disease

## Data Availability

No new data were created or analyzed in this study.
